# Six-month humoral immune response to inactivated COVID-19 vaccine among people living with HIV

**DOI:** 10.3389/fimmu.2022.988304

**Published:** 2022-10-17

**Authors:** Shi Zou, Wei Guo, Songjie Wu, Fangzhao Ming, Yuting Tan, Mengmeng Wu, Weiming Tang, Ke Liang

**Affiliations:** ^1^ Department of Infectious Diseases, Zhongnan Hospital of Wuhan University, Wuhan, China; ^2^ Wuhan Research Center for Infectious Diseases and Cancer, Chinese Academy of Medical Sciences, Wuhan, China; ^3^ Department of Pathology, Zhongnan Hospital of Wuhan University, Wuhan, China; ^4^ Department of Pathology, School of Basic Medical Sciences, Wuhan University, Wuhan, China; ^5^ Department of Nosocomial Infection Management, Zhongnan Hospital of Wuhan University, Wuhan, China; ^6^ Wuchang District Center for Disease Control and Prevention, Wuhan, China; ^7^ Guangdong Second Provincial General Hospital, Guangdong, China; ^8^ The University of North Carolina at Chapel Hill Project-China, Guangzhou, China; ^9^ Hubei Engineering Center for Infectious Disease Prevention, Control and Treatment, Wuhan, China

**Keywords:** neutralizing antibodies, inactivated COVID-19 vaccine, people living with HIV (PLWH), seroconversion, longitudinal humoral response

## Abstract

Longitudinal humoral immune response to inactivated COVID-19 vaccines among people living with HIV (PLWH) have not yet been systematically investigated. We conducted a 6-month longitudinal study among vaccinated PLWH and HIV-Negative Controls (HNC) to determine whether the humoral immune response effects of the inactivated COVID-19 vaccine are different between the two groups of people. Totally, 46 PLWH and 38 HNC who received the inactivated COVID-19 vaccine on days 0 and 28 were enrolled. The SARS-CoV-2 neutralizing antibodies (nAbs) and total specific IgM and IgG antibodies were examined on Day 0-Day190. The level and positive seroconversion rate of nAbs peaked on Day 42 in HNC while peaked on Day 70 in PLWH, then decreased gradually with the extension of the vaccination period after the peaks. The peak level of nAbs in PLWH on Day 70, (GMC 8.07 BAU/mL, 95% CI 5.67-11.48) was significantly lower than in HNC on Day 42 (GMC 18.28 BAU/mL, 95% CI 10.33-32.33, P =0.03). The decrease in the geometric mean concentrations (GMCs) of nAbs was observed as 42.9% in PLWH after peak level, which decreased from 8.07 BAU/mL [95% CI: 5.67-11.48] on Day 70 to 4.61 BAU/mL [95% CI: 3.35-6.34] on Day 190 (p = 0.02). On Day 190, only seven (18%, [95% CI: 6-40]) HNC and five (11%, [95% CI: 4-25]) PLWH maintained positive nAbs response respectively. The geometric mean ELISA units (GMEUs) and positive seroconversion rate of IgG in PLWH dropped significantly from Day 70 (GMEUs, 0.20 EU/mL, [95% CI: 0.13-0.34]; seroconversion, 52%, [95% CI: 34-69]) to Day 190 (GMEUs, 0.05 EU/mL, [95% CI: 0.03-0.08], P<0.001; seroconversion, 18%, [95% CI: 8-33], P<0.001). There was no significant difference in levels and seroconversion rates of nAbs and IgG between the two groups on Day 190. The peak immunogenicity of the inactivated COVID-19 vaccine was delayed and inferior in PLWH compared to HNC, while no significant difference was found in six-month immunogenicity between the two groups.

## Introduction

Coronavirus disease 2019 (COVID-19) caused by Severe Acute Respiratory Syndrome Coronavirus 2 (SARS-CoV-2) represents an unprecedented public health, social and economic challenge (1). Safe and effective vaccines are therefore necessary to control COVID-19. In China, inactivated vaccines such as BBIBP-CorV (Sinopharm), WIBP-CorV (Sinopharm), and CoronaVac (Sinovac Biotech) are widely used (2). However, the data on the immune effectiveness of the inactivated COVID-19 vaccines among immunocompromised groups, such as people living with HIV (PLWH), are largely missing. These missing data greatly restrict the progress of vaccination in China (3).

Both the United Nations AIDS program (UNAIDS) and Chinese national guidelines recommend COVID-19 vaccination for PLWH regardless of their immune status (4, 5). However, full immune reconstitution might not be possible in PLWH even if the virus is suppressed (6, 7), raising concerns about the suboptimal responses (weaker humoral or cellular immune response) of COVID-19 vaccines in PLWH. Previous studies of various COVID-19 vaccines suggested that there was no statistical difference in humoral or cellular immune responses between PLWH who have viral suppression and high CD4^+^ T lymphocyte count (CD4 count > 200 cells/µL) and HIV-Negative Controls (HNC) (8–10). Several studies about the inactivated COVID-19 vaccines showed that the immunogenicity in PLWH with CD4 count < 200/μL was lower compared to HNC (11, 12). Nevertheless, all these data were from short-term or cross-sectional studies to BBIBP-CorV or CoronaVac vaccine, while none of them monitored the change of longitudinal immune responses to WIBP-CorV vaccine among PLWH.

Our previous study demonstrated that early humoral immune response (Day 70) to this inactivated COVID-19 vaccine was delayed in PLWH who are stable on antiretroviral therapy (ART) with unsuppressed CD4 count than that in HNC (13). Moving forward, this study aimed to further investigate the longitudinal humoral immune response to the inactivated COVID-19 vaccine up to six months from the first dose in PLWH, comparing it with HNC.

## Materials and methods

### Study design and participants

Between March and December 2021, 48 PLWH and 40 HNC aged 18 to 59 years were enrolled for this study. All participants had no history of SARS-CoV-2 infection (via serological and nucleic acid test). They were all given the inactivated COVID-19 vaccine (Sinopharm, WIBP-CorV, Wuhan Institute of Biological Products Co. Ltd, Wuhan, China) in the Wuchang district of Wuhan, China, on Day 0 and Day 28. Our analysis excluded two PLWHs and two HNC who were missing at the prescribed study timelines. Finally, 46 PLWH and 38 HNC were enrolled in this study. Peripheral blood samples were collected at baseline (Day 0), Day 14, Day 28, Day 42, Day 70, Day 100, Day 130, Day 160, and Day 190. The CD4 count of all participants was tested at baseline. Clinical and laboratory data regarding the HIV status of PLWH were obtained from the China National HIV/AIDS Comprehensive Response Information Management System (CRIMS).

### SARS-CoV-2 antibody testing

The nAbs and the specific IgM and IgG-binding antibody responses to SARS-CoV-2 were measured at each time point. An in-house SARS-CoV-2 nAbs assay kit by surrogate virus neutralization test (Zhuhai Livzon Diagnostics Inc, Zhuhai, China) was used to examine the serum level of nAbs against the spike protein receptor-binding domain (RBD) according to instructions of the manufacturers (13). An in-house semi-quantitative ELISA kit (Livzon) was used to examine the serum levels of the total specific IgM and IgG antibodies (12). We defined the positive responses of nAbs, IgM, and IgG as ≥ 10 BAU/mL, 0.15 EU/mL, and 0.18 EU/mL, respectively. Seroconversion of antibodies was defined as a change from baseline seronegative to seropositive. All test kits used were approved by the China Food and Drug Administration and provided by Zhuhai Livzon Diagnostics Inc.

### Statistical analysis

Categorical variables were presented as n (%) and compared using the Chi-square test or Fisher’s exact test. T-test or the Mann-Whitney U test (for nonnormally distributed data) was used to analyze antibody geometric mean concentrations or ELISA units between PLWH and HNC groups. 95% CIs for all categorical outcomes were calculated using the Clopper-Pearson method. Statistical significance was defined as a two-sided P-value of less than 0.05. All statistical analyses were conducted using SPSS (Version 26). We defined seroconversion of antibodies as a change from baseline seronegative to seropositive. Immune response was defined as the reaction of the body to the presence of an antigen. And immunogenicity was defined as the property of eliciting an immune response.

## Results

### Characteristics of the enrolled individuals

This study was a continuation of our previous study about the early humoral immune response (Day 70) to the inactivated COVID-19 vaccine in PLWH (13). Characteristics of the 46 PLWH and 38 HNC are shown in **Table 1**. 87% (40/46) PLWH were male, and the median (IQR) age of PLWH was 36 (31–42) years. All PLWH were receiving ART, and 89% (41/46) had virus suppressed (HIV viral load < 50 copies/mL). The median CD4 count of the PLWH and HNC was 523 (IQR: 351-653) and 675 (IQR: 540-828)/μL, respectively.

**Table 1:** Baseline characteristics of the study participants in Wuhan, China (N = 84), 2021.

Characteristics	HNCs group (n = 38)	PLWH group (n = 46)	P-value
**Age in years, median (IQR)**	31 (27–39)	36 (31-42)	0.064
**Men, No. (%)**	19 (50)	40 (87)	<0.001
**HIV-VL<50 copies/mL, No. (%)**	/	41 (89)	/
**ART regimens**			
** No, n(%)**	/	0 (0)	/
** NNRTI based**	/	38 (83)	/
** Integrase inhibitor based**	/	7 (15)	/
** PIs based**	/	1 (2)	/
**CD4 count (cells/μL), median** ** <200, No. (%)** ** 200-499, No. (%)** ** >500, No. (%)**	675 (540, 828) 0 (0) 0 (0) 38 (100)	523 (351, 653) 2 (4) 19 (41) 25 (55)	0.002 / / <0.001

Data are n (%), Standard Deviation (SD) or median (IQR). Data are for participants with HIV and without HIV included in this analysis. NNRTI (Non-Nucleoside Reverse Transcriptase Inhibitor): efavirenz and nevirapine were used in our study. Integrase inhibitor: dolutegravir and bictegravir were used. PIs: only lopinavir/ritonavir were used in our study.

### Neutralizing antibody responses to vaccination

**Figure 1:**
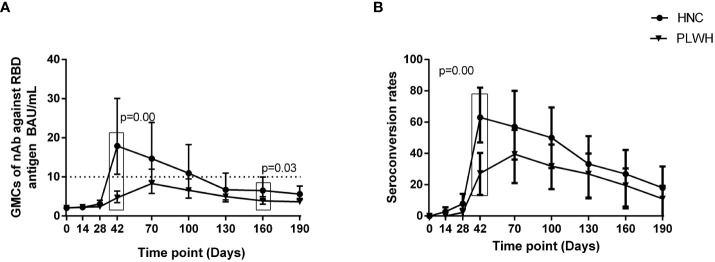
Neutralizing antibody responses to vaccination with inactivated COVID-19 vaccine among PLWH and HNC. Titers **(A)** and seroconversion rates **(B)** of Neutralizing antibody against RBD antigen at days 0-190 after vaccination. The threshold for a positive response is shown by the hashed line at 10 BAU/mL. Data points are medians (error bars represent 95% CI). P value calculated using Mann–Whitney U test **(A)** or Fisher’s exact test **(B)**.

Our previous study demonstrated that nAbs response to the inactivated COVID-19 vaccine was delayed and lower in PLWH than in HNC (13). The GMCs were significantly lower in PLWH on peak Day 70 than in HNC on peak Day 42 (p=0.03), while the seroconversion rates of the two groups were similar (p=0.07, **Figures 1A, B**). The nAbs GMCs decreased gradually with the extension of the vaccination period in each group after the peak. The decrease in the GMCs of nAbs was observed as 42.9% in PLWH after peak level (Day 70: 8.07 BAU/mL, [95% CI: 5.67-11.48] versus Day 190: 4.61 BAU/mL [95% CI: 3.35-6.34]; p = 0.02; **Figure 1A**). Similar to GMCs, the seroconversion rate of nAbs in PLWH on Day 190 (11%, [95% CI: 4%-25%]) was also significantly lower than that on Day 70 (39%, [95% CI: 24%-58%]; p = 0.00). There was no significant difference in GMCs and seroconversion rate of nAbs between the two groups on Day 190.

### Binding-antibody responses to vaccination

**Figure 2:**
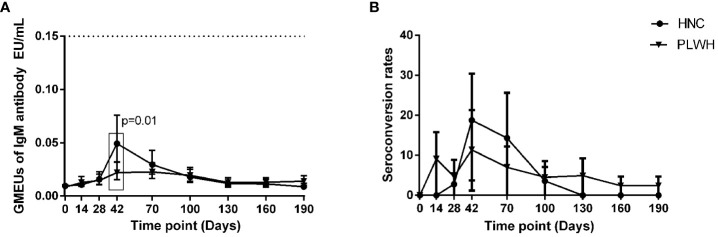
IgM antibody responses to vaccination with inactivated COVID-19 vaccine in PLWH and HNC. Titers **(A)** and seroconversion rates **(B)** of IgM at days 0-190 after vaccination. The threshold for a positive response is shown by the hashed line at 0.15 EU/mL. Data points are medians (error bars represent 95% CI). P value calculated using Mann–Whitney U test **(A)** or Fisher’s exact test **(B)**.

**Figure 3:**
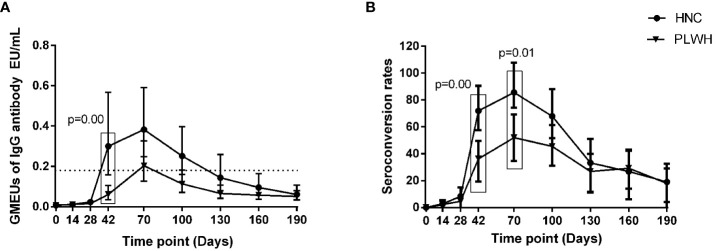
IgG antibody responses to vaccination with inactivated COVID-19 vaccine in PLWH and HNC. Titers **(A)** and seroconversion rates **(B)** of IgG at days 0-190 after vaccination. The threshold for a positive response is shown by the hashed line at 0.18EU/mL. Data points are medians (error bars represent 95% CI). P value calculated using Mann–Whitney U test **(A)** or Fisher’s exact test **(B)**.

The geometric mean ELISA units (GMEUs) and positive seroconversion rate of IgM in both groups were consistently low during the observation period (**Figures 2A, B**). The GMEUs and positive seroconversion rate of IgG in both groups peaked on Day 70 and decreased gradually with the extension of the vaccination period (**Figure 3A, B**). There was no difference in the peak (Day 70) GMEUs of IgG between the two groups, while the peak (Day 70) positive seroconversion rate of IgG in PLWH were significantly lower than that in HNC (P = 0.01). As expected, the GMEUs and positive seroconversion rate of IgG in PLWH dropped significantly on Day 190 (GMEUs, 0.05 EU/mL, [95% CI: 0.03-0.08]; seroconversion, 18%, [95% CI: 8%-33%]) compared with Day 70 (GMEUs, 0.20 EU/mL, [95% CI: 0.13-0.34], P = 0.00; seroconversion, 52%, [95% CI: 34%-69%], P = 0.00). There was no significant difference in GMEUs and seroconversion rate of IgG between the two groups on Day 190.

Then the association between IgG and nAbs in PLWH was analyzed. We observed a strong and consistent positive correlation from Day 28 to Day 190 (p < 0.05). Age, sex, comorbidities, and CD4 count were not associated with the levels and positive seroconversion rate of nAbs and IgG seroconversion at all time points in PLWH.

## Discussion

It is essential to monitor the change in immune response among PLWH over time. In the present study, we corroborated and extended our previous results by reporting the humoral immune response in PLWH after two doses of inactivated COVID-19 vaccine. Our results fill the knowledge gap concerning immune responses to SARS-CoV-2 vaccines among PLWH who are in countries that mainly use inactivated SARS-CoV-2 vaccines.

Our previous study revealed that the positive conversion rate of the SARS-CoV-2 specific antibodies was relatively lower and quickly lost in PLWH with COVID-19 (14), raising concerns about the poor immunogenicity of COVID-19 vaccine in PLWH. Some case reports also showed specific SARS-CoV-2 antibodies response were delayed or even vanished in PLWH with COVID-19 (15). This study further confirmed these findings, which indicated that the peak time of immunogenicity in PLWH was 1 month later than that in HNC, suggesting PLWH need to take longer to develop a humoral immune response to the inactivated COVID-19 vaccine. A study about mRNA vaccine against SARS-CoV-2 in immunocompromised patients demonstrated that whether humoral, cellular, or both immune responses are delayed depends on the patient group, therapy, and individual risk factors (16). The factors that delay the immune responses to the inactivated COVID-19 vaccine in PLWH are needed to be further explored.

Our results were consistent with previous studies showing that PLWH had a lower immune response to inactivated COVID-19 vaccines than HNC (11, 17). Studies by cross-sectional analysis showed that the level of nAbs in PLWH was lower than the levels in HNC at different time points after the second dose of the inactivated COVID-19 vaccination (17, 18). However, no studies have compared peak humoral immune response in PLWH and HNC groups. In our study, the level of nAbs and the positive seroconversion rate of IgG in PLWH at peak time were significantly lower than in HNC at peak time. The level and positive seroconversion rate of nAbs and IgG declined in our study over time in either PLWH or HNC. The decrease in the levels of nAbs and IgG were observed as 42.9% and 75% in PLWH between Day 70 and Day 190, respectively. Considering nAbs and special antibodies could collaborate to neutralize and eliminate the virus (19, 20), more methods should be taken to maintain humoral immunity to SARS-CoV-2 after vaccination (such as a third dose).

Antibody responses to mRNA, adenovirus vector, and inactivated COVID-19 vaccines all demonstrated that lower CD4 count (<200/μl) or detectable viral loads were the risk factors for lower nAbs levels among PLWH (18, 21, 22). However, the humoral immune response was more inadequate in our PLWH participants (all of whom were on ART and the majority had virus suppressed and CD4 count >350/μl) regardless of their CD4 count or HIV viral loads. And no correlation between antibody responses and CD4 count in all-time points was found in our study. Although clinical management and effective ART have improved long-term outcomes for PLWH, residual inflammation on ART and ongoing immune dysregulation among PLWH may influence the effects of the vaccines (23).

Of course, our study is not exempt from limitations. First, T-cell immunity was not assessed. As the cellular immune response is a key player in the active protection from severe COVID-19, further studies are needed to be followed to assess the cellular immune response of the inactivated COVID-19 vaccine in PLWH. Second, our PLWH sample included mostly male patients with a CD4 count >350/μL. Therefore, the vaccine immunogenicity among PLWH with unsuppressed viremia or advanced immunosuppression needs further investigation. A previous study showed equivalent responses in males and females using this vaccine (24), which may mitigate some of the sex imbalance in this study. Third, the best correlation of antibody responders in PLWH with normal CD4 count is currently unknown, and more large-scale population studies are needed.

In summary, after the peaks, the humoral immune response decreased gradually with the extension of the vaccination period in both the PLWH and HNC groups. Although there was no significant difference in six-month immunogenicity between the two groups, the peak immunogenicity of the inactivated COVID-19 vaccine was delayed and lower in PLWH. Therefore, more concern should be taken on this special population.

## Data availability statement

The original contributions presented in the study are included in the article/supplementary material. Further inquiries can be directed to the corresponding authors.

## Ethics statement

The study was approved by the Research and Ethics Committee of Zhongnan Hospital, Wuhan University, P. R. China (2020079K-1). Informed consent was obtained from all individuals enrolled in this study, and the study was done in accordance with the principles of the Declaration of Helsinki and Good Clinical Practice. The patients/participants provided their written informed consent to participate in this study.

## Author contributions

WT and KL conceived and designed this investigation. SZ, MW, and FM helped to design the scheme of the investigation. SZ, MW, and FM collected the original data. WG, YT, and SW analyzed the data. WT and KL contributed to the interpretation of the data. SZ, WG, WT, and KL contributed to the writing of the paper. All authors contributed to the article and approved the submitted version.

## Funding

This work was supported by the National Key Research and Development Program of China (2017YFE0103800), the National Nature Science Foundation of China (81903371), NIMH (R34MH119963), the National Science and Technology Major Project (2018ZX10101-001-001-003), Shenzhen Fundamental Research Program (JCYJ20210324140401004), Special Found on Prevention and Control of New Coronary Pneumonia in Guangdong Universities (2020KZDZX1047), Medical Science and Technology Innovation Platform Support Project of Zhongnan Hospital, Wuhan University (PTXM2020008), the Non-profit Central Research Institute Fund of Chinese Academy of Medical Sciences (2020-PT320-004), Science and Technology Innovation Cultivation Fund of Zhongnan Hospital, Wuhan University (cxpy2017043), Medical Science Advancement Program (Basic Medical Sciences) of Wuhan University (TFJC2018004), and Discipline Cultivation Project of Department of Infectious Diseases, Zhongnan Hospital, Wuhan University (ZNXKPY2021027).

## Conflict of interest

The authors declare that the research was conducted in the absence of any commercial or financial relationships that could be construed as a potential conflict of interest.

## Publisher’s note

All claims expressed in this article are solely those of the authors and do not necessarily represent those of their affiliated organizations, or those of the publisher, the editors and the reviewers. Any product that may be evaluated in this article, or claim that may be made by its manufacturer, is not guaranteed or endorsed by the publisher.
