# Corrigendum: Six-month humoral immune response to inactivated COVID-19 vaccine among people living with HIV

**DOI:** 10.3389/fimmu.2024.1494940

**Published:** 2024-09-25

**Authors:** Shi Zou, Wei Guo, Songjie Wu, Fangzhao Ming, Yuting Tan, Mengmeng Wu, Weiming Tang, Ke Liang

**Affiliations:** ^1^ Department of Infectious Diseases, Zhongyang Hospital of Wuhan University, Wuhan, China; ^2^ Wuhan Research Center for Infectious Diseases and Cancer, Chinese Academy of Medical Sciences, Wuhan, China; ^3^ Department of Pathology, Zhongnan Hospital of Wuhan University, Wuhan, China; ^4^ Department of Pathology, School of Basic Medical Sciences, Wuhan University, Wuhan, China; ^5^ Department of Nosocomial Infection Management, Zhongnan Hospital of Wuhan University, Wuhan, China; ^6^ Wuchang District Center for Disease Control and Prevention, Wuhan, China; ^7^ Guangdong Second Provincial General Hospital, Guangdong, China; ^8^ The University of North Carolina at Chapel Hill Project-China, Guangzhou, China; ^9^ Hubei Engineering Center for Infectious Disease Prevention, Control and Treatment, Wuhan, China

**Keywords:** neutralizing antibodies, inactivated COVID-19 vaccine, people living with HIV (PLWH), seroconversion, longitudinal humoral response

In the published article there was an error in affiliation 7. Instead of “Guangdong No. 2 Provincial People’s Hospital, Guangzhou, China”, it should be “Guangdong Second Provincial General Hospital, Guangdong, China”.

In the published article, there was an error in the **Funding** statement. One of the funding sources was not included (Shenzhen Fundamental Research Program (JCYJ20210324140401004)). The correct **Funding** statement appears below.

“This work was supported by the National Key Research and Development Program of China (2017YFE0103800), the National Nature Science Foundation of China (81903371), NIMH (R34MH119963), the National Science and Technology Major Project (2018ZX10101-001-001-003), Shenzhen Fundamental Research Program (JCYJ20210324140401004), Special Found on Prevention and Control of New Coronary Pneumonia in Guangdong Universities (2020KZDZX1047), Medical Science and Technology Innovation Platform Support Project of Zhongnan Hospital, Wuhan University (PTXM2020008), the Non-profit Central Research Institute Fund of Chinese Academy of Medical Sciences (2020-PT320-004), Science and Technology Innovation Cultivation Fund of Zhongnan Hospital, Wuhan University (cxpy2017043), Medical Science Advancement Program (Basic Medical Sciences) of Wuhan University (TFJC2018004), and Discipline Cultivation Project of Department of Infectious Diseases, Zhongnan Hospital, Wuhan University (ZNXKPY2021027).”

The authors apologize for these errors and state that they do not change the scientific conclusions of the article in any way. The original article has been updated.

